# Integration of multicopy extrachromosomal transgenes into defined loci without phenotypes

**DOI:** 10.17912/micropub.biology.000571

**Published:** 2022-05-18

**Authors:** Sawako Yoshina, Shohei Mitani

**Affiliations:** 1 Department of Physiology, Tokyo Women’s Medical University School of Medicine, Tokyo 162-8666, Japan.

## Abstract

We show how presumably non-phenotypic loci can be used for integration sites of multi-copy extrachromosomal transgenes, using the CRISPR/Cas9 system. We used four loci, which show no apparent phenotype in our hands, as a model for any other loci with no phenotype.

**Figure 1. Isolation of multicopy integrant strains by the CRISPR/Cas9 system.**
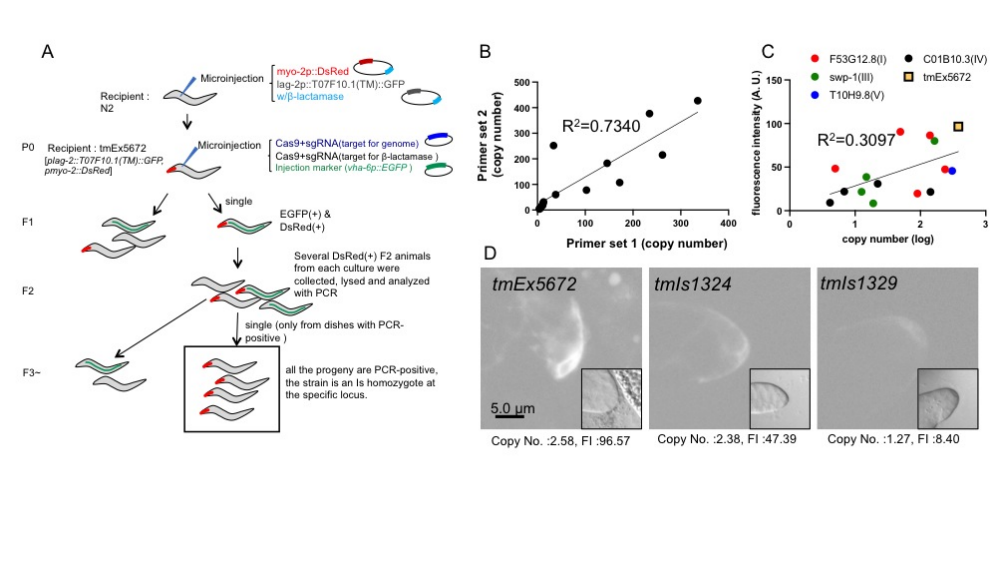
(A) Procedures for isolation of multicopy integrant strains by the CRISPR/Cas9 system. Fluorescent markers are shown by green (EGFP) and red (DsRed). (B) Relative amounts of the *lag-2* promoter and the *T07F10.1* gene as determined by quantitative PCR. Primer set 1 is targeted at the *lag-2* promoter. Primer set 2 is targeted at the *T07F10.1* gene. The data were normalized with the *ama-1* gene. The copy numbers are presented as a ratio to the wild-type N2 (n>3). (C) A scatter plot of the copy number of integrated sequences (abscissa) against fluorescence intensity (ordinate) in the *Ex5672* , and the 14 *Is* lines. The X axis is shown in log scale. The graph represents the intensity of fluorescence of DTC (y-axis, n > 20 animals per strain). Fluorescence intensity was measured using ImageJ (NIH, Bethesda, MD). (D) Example of GFP protein expressed in DTC. Scale bar = 5.0 µm. (2 sec. exposure time, gain X 4)

## Description

In previous work from our laboratory we showed that extrachromosomal transgenes can be integrated into genome loci using the CRISPR/Cas9 system (Yoshina *et al.* , 2016). The method is very useful because expression of transgenes can be dependent on the integration sites and researchers typically integrated extrachromosomal transgenes into random loci by gamma irradiation (Way *et al* ., 1991), UV irradiation (Mitani, 1995), microparticle bombardment (Praitis et al. 2001, Radman et al., 2013), mini Singlet Oxygen Generator (Noma and Jin, 2018), or TMP/UV (Kage-Nakadai *et al.* , 2012). A transgenic strain in which stably high expressing transgenes (e.g. Cre recombinase) are integrated into the desired chromosome is convenient for crossing with other gene modified strains (Kage-Nakadai *et. al.* , 2014). It is also useful if one wants to study the effect of expression level by making distinct insertions that express at different levels by changing copy number in the integrated array.

To carry out such functional analyses, researchers sometimes need to integrate extrachromosomal transgenes into loci that rarely interfere with the functional analyses. In our previous work, we demonstrated the integration of extrachromosomal DNA into two loci, *dpy-3* and *ben-1* , allowed us to readily identify integrant strains. Both loci show some phenotypes, especially for the *dpy-3* locus. In the present report, we show how presumably non-phenotypic loci can also be used for integration sites. We used the *F53G12.8(I; -19.52),* *swp-1(III; -1.45), C01B10.3(IV; 3.22)* and *T10H9.8(V; 0.13)* loci, which show no apparent phenotype in our hands, as a model for other loci with no phenotype. We presume that many other loci will also work.

We injected plasmids with P0 extrachromosomal transgenic animals as shown in Fig. 1A. The number of P0 animals used are as follows: *F53G12.8* -specific sgRNA, 50 animals *;* *swp-1-* specific sgRNA, 69 animal; *C01B10.3* -specific sgRNA, 107 animals *;* and *T10H9.8-* specific sgRNA, 82 animals.

After F1 animals hatched, we singled EGFP and DsRed double positive F1 animals. The number of single cultured F1 animals are as follow: *F53G12.8* -specific sgRNA, 69 animals; *swp-1-* specific sgRNA, 112 animals; *C01B10.3* -specific sgRNA, 154 animals and *; T10H9.8-* specific sgRNA, 140 animals.

Among the dishes with DsRed(+) F2 animals, we picked more than five animals from each dish; selected animals are supposed to be multicopy-positive but injection marker-negative, which enabled us to isolate weak fluorescence transgenic strains. We then performed PCR as described in the Methods.

We generated five strains at the *F53G12.8* locus ( *tmIs1323, tmIs1325, tmIs1324, tmIs1322, tmIs1333* ), 4 strains at the *swp-1* locus ( *tmIs1329, tmIs1330, tmIs1335, tmIs1337),* 5 strains at the *C01B10.3* locus ( *tmIs1331, tmIs1332, tmIs1336, tmIs1340, tmIs1341.* Since homozygotes of *tmIs1341* have a Let phenotype, we did not use this strain in this study), and 3 strains at the *T10H9.8(V)* locus ( *tmIs1326, tmIs1327/+, tmIs1338/+* . Since homozygotes of *tmIs1327* and *tmIs1338* have a Let phenotype, we did not use these two strains in this study). We performed PCR to determine if the transgenic lines carried Cas9 gene. As a result, *tmIs1337* was found to carry the complete Cas9 gene. Thus, the integration efficiencies (strains / F1 animals) for loci examined were 5/69, 4/112, 5/154, 3/140 for *F53G12.8* , *swp-1* , *C01B10.3* and *T10H9.8* , respectively.

To determine the copy number of insertions, we performed quantitative PCR using genomic DNA as a template. We designed primer sets located within the promoter of the *lag-2* gene and the *T07F10.1* gene that were contained in the extrachromosomal array and in the *C. elegans* genome (Fig. 1B). The parent *Ex* line and integrant strains were tested and compared to the wild-type N2. There was a positive correlation between the values obtained from the two primer sets (R ^2^ =0.7340, Fig. 1B), suggesting that two primer sets show reproducible values. We compared the average of the qPCR values obtained from the two primer sets with the fluorescence intensity at the DTC (Fig. 1C, D). As a result, there was a positive correlation between copy number of integrated sequences and fluorescence intensity (R ^2^ =0.3097, Fig. 1C, D). We used integration sites of four chromosomes at the central regions or peripheral region of an arm. The chromosomal positions of the integration sites had no apparent effect on the fluorescence intensity/copy number ratio.

As shown in the present paper, we could easily isolate locus-specific integrants without phenotype selection. Because multicopy transgenes are sometimes useful for overexpression of endogenous or exogenous genes, integrant strains are valuable for functional analyses. Integrant lines support stable expression of transgenes compared with extrachromosomal lines that show mosaic expression with loss of transgene during cell division. However, because conventional multicopy transgenes usually have randomly rearranged structures without any information on integration sites by simple PCR experiments unlike single-copy integration experiments (Frøkjær-Jensen *et. al.,* 2008). Thus, this method is expected to isolate multicopy integrant transgenic strains to analyze different gene expression levels by changing copy number in the integrated array. If we can choose a few loci where arrays can be inserted efficiently, researchers are able to plan the combination of transgenic and mutant alleles.

## Methods

Construction of plasmids

We used the plasmid, *lag-2p::T07F10.1(TM)::GFP* , as previously described (Yoshina *et al* ., 2012). The oligonucleotides used for sgRNA-specific sequences against *swp-1* were forward (5’-ACGTGGATTAGAGGCAGGTTTTAGAGCTAGAAATAGC-3’) and reverse (5’-GCCTCTAATCCACGTTGCAAGACATCTCGCAATAGGAGG-3’). We used the sgRNA-specific sequences against *F53G12.8, C01B10.3* and *T10H9.8* as previously described (Dejima *et al* ., 2018).

Generation of integrant strains

As depicted in Fig. 1a as a schematic drawing of experimental procedure, integrant strains are isolated. Worms with an extrachromosomal transgene of interest ( *tmEx5672* ) are generated by microinjection of a plasmid ( *myo-2p::DsRed* with conventional ampicillin-resistant backbone) to be integrated by a standard method (Mello *et al* ., 1991). P0 animals with extrachromosomal array are injected with Cas9 and sgRNA plasmids for the integration site, and sgRNA for the beta-lactamase gene and an injection marker ( *vha-6p::EGFP* ). The plasmid concentration was used as follows. sgRNA for integration site : sgRNA for beta-lactamase : *vha-6p::EGFP* : pBlueScript = 1:1:5:15. F1 animals are picked up: To obtain integrated lines, EGFP, DsRed double positive F1 are singled to new plates. After F2 animals are born, more than 5 DsRed(+) animals from each plate are collected and used for PCR. We performed PCR with 4 different primer sets for identification of the integrated strains. The primer sets we used are as follows.

Set A : locus specific primer#Fwd and beta-lactamase specific primer : 5’-GAGCTGAATGAAGCCATACCA-3’.

Set B: locus specific primer#Fwd and beta-lactamase specific primer : 5’-GTAGATAACTACGATACGGGA-3’.

Set C : locus specific primer#Rev and beta-lactamase specific primer : 5’-GAGCTGAATGAAGCCATACCA-3’.

Set D: locus specific primer#Rev and beta-lactamase specific primer : 5’-GTAGATAACTACGATACGGGA-3’.

*F53G12.8* locus specific primer#Fwd : 5’-GTTCAGTCTTGGTTGCCCAGG-3’

*F53G12.8* locus specific primer#Rev : 5’-TCGGCGGATTCGGCAACTGA-3’

*swp-1* locus specific primer#Fwd : 5’-AGGAATGACCAACGAAATGC-3’

*swp-1* locus specific primer#Rev : 5’-TTCAGCCTTCTTCCGTGTCT-3’

*C01B10.3* locus specific primer#Fwd : 5’-TCGAGTTCTCTCAACAGTGG-3’

*C01B10.3* locus specific primer#Fwd : 5’-GATGGAACTCGTGATTTGGC-3’

*T10H9.8* locus specific primer#Fwd : 5’-TGTACCATGACCTCCAGGAG-3’

*T10H9.8* locus specific primer#Rev : 5’-TCGTGTCCTGTAGGATTTCGC-3’

If DNA amplification was seen in any of the above 4 PCR sets, we considered a candidate for an integrated animal. The amplified DNA was confirmed by Sanger sequencing and we determined that it is an integrated animal.

After identifying the successful integration strains, which may include heterozygous strains, DsRed(+) F2 or later generation animals are singled more than 8 candidate animals to fresh dishes. When we find dishes with all animals are DsRed-positive, the animals are homozygote integrant lines.

Quantitative PCR

Quantitative PCR was performed in a 7500 Real-time Thermal cycler (Applied Biosystems) using the Power SYBR master mix (Applied Biosystems) with the following parameters: 95˚C for 10 min and 40 cycles of 95˚C for 5 s, 60˚C for 10 s and 72˚C for 34 s. All data were normalized to the *ama-1* gene. A component of the *lag-2* promoter was amplified by using the primers 5'-TTGTCAGTCGCTGCAAGAAC-3' and 5'-TGTGCAAAGTGTGTCCAACA-3'. A component of the *T07F10.1* was amplified by using the primers 5'-ATGGCTCATTCCCTCAGATG-3' and 5'-TGTGCCATGTGATAAATGCTG-3'.

## Reagents

**Table:** 

Strain	Genotype	Available from
N2	*Caenorhabditis elegans*	CGC
*tmEx5672*	pFX_ *myo-2p::DsRed* , *lag-2p::T07F10.1(TM)::GFP*	Mitani Lab
*tmIs1322*	pFX_ *myo-2p::DsRed* , *lag-2p::T07F10.1(TM)::GFP*	Mitani Lab
*tmIs1323*	pFX_ *myo-2p::DsRed* , *lag-2p::T07F10.1(TM)::GFP*	Mitani Lab
*tmIs1324*	pFX_ *myo-2p::DsRed* , *lag-2p::T07F10.1(TM)::GFP*	Mitani Lab
*tmIs1325*	pFX_ *myo-2p::DsRed* , *lag-2p::T07F10.1(TM)::GFP*	Mitani Lab
*tmIs1326*	pFX_ *myo-2p::DsRed* , *lag-2p::T07F10.1(TM)::GFP*	Mitani Lab
*tmIs1327*	pFX_ *myo-2p::DsRed* , *lag-2p::T07F10.1(TM)::GFP*	Mitani Lab
*tmIs1329*	pFX_ *myo-2p::DsRed* , *lag-2p::T07F10.1(TM)::GFP*	Mitani Lab
*tmIs1330*	pFX_ *myo-2p::DsRed* , *lag-2p::T07F10.1(TM)::GFP*	Mitani Lab
*tmIs1331*	pFX_ *myo-2p::DsRed* , *lag-2p::T07F10.1(TM)::GFP*	Mitani Lab
*tmIs1332*	pFX_ *myo-2p::DsRed* , *lag-2p::T07F10.1(TM)::GFP*	Mitani Lab
*tmIs1333*	pFX_ *myo-2p::DsRed* , *lag-2p::T07F10.1(TM)::GFP*	Mitani Lab
*tmIs1335*	pFX_ *myo-2p::DsRed* , *lag-2p::T07F10.1(TM)::GFP*	Mitani Lab
*tmIs1336*	pFX_ *myo-2p::DsRed* , *lag-2p::T07F10.1(TM)::GFP*	Mitani Lab
*tmIs1337*	pFX_ *myo-2p::DsRed* , *lag-2p::T07F10.1(TM)::GFP*	Mitani Lab
*tmIs1338*	pFX_ *myo-2p::DsRed* , *lag-2p::T07F10.1(TM)::GFP*	Mitani Lab
*tmIs1340*	pFX_ *myo-2p::DsRed* , *lag-2p::T07F10.1(TM)::GFP*	Mitani Lab
*tmIs1341*	pFX_ *myo-2p::DsRed* , *lag-2p::T07F10.1(TM)::GFP*	Mitani Lab
		
Plasmid	Genotype	Description
pDD162	Cas9-sgRNA vector	Addgene
pFX_ *vha-6p::EGFP*	*vha-6p::EGFP*	Promoter of 1129 bp DNA fragment was fused to EGFP cDNA.
pFX_ *myo-2p::DsRed*	*myo-2p::DsRed*	Promoter of 1040 bp DNA fragment was fused to DsRed cDNA.
*lag-2p::T07F10.1(TM)::GFP*	*T07F10.1(TM)::GFP*	Promoter of 3000 bp DNA fragment was fused to *T07F10.1* (150 bp) and GFP cDNA.
